# The Effect of Virtual Reality Training on Anticipatory Postural Adjustments in Patients with Chronic Nonspecific Low Back Pain: A Preliminary Study

**DOI:** 10.1155/2021/9975862

**Published:** 2021-07-27

**Authors:** Zhicheng Li, Qiuhua Yu, Haizhen Luo, Wenzhao Liang, Xin Li, Le Ge, Siyun Zhang, Le Li, Chuhuai Wang

**Affiliations:** ^1^Department of Rehabilitation Medicine, The First Affiliated Hospital, Sun Yat-sen University, Guangzhou 510080, China; ^2^Department of Radiology, The First Affiliated Hospital, Sun Yat-Sen University, Guangzhou 510080, China; ^3^Institute of Medical Research, Northwestern Polytechnical University, Xi'an 710072, China

## Abstract

**Objectives:**

This study is aimed at exploring the effects of virtual reality (VR) training on postural control, measured by anticipatory and compensatory postural adjustments (APAs and CPAs, respectively), in patients with chronic nonspecific low back pain (CNLBP) and the potential neuromuscular mechanism of VR training.

**Methods:**

Thirty-four patients were recruited and randomly assigned to the VR group (*n* = 11), the motor control exercise group (MCE, *n* = 12) and the control group (CG, *n* = 11). The VR group received VR training using Kinect Xbox 360 systems and magnetic therapy. Besides magnetic therapy, the participants in the MCE group performed real-time ultrasound-guided abdominal drawing-in maneuver (ADIM) and four-point kneeling exercise. The CG only received magnetic therapy. Surface muscle electromyography (sEMG) was used to record the muscle activities of transverse abdominis (TrA), multifidus (MF), lateral gastrocnemius (LG), and tibialis anterior (TA) during ball-hitting tasks. The muscle activation time and integrals of the electromyography activities (IEMGs) during the APA and CPA stages were calculated and used in the data analysis. The visual analogue scale (VAS) and Oswestry dysfunction index (ODI) scores were also recorded.

**Results:**

A significant interaction effect of time × group was observed on the activation time of TrA (*p* = 0.018) and MF (*p* = 0.037). The post-intervention activation time of the TrA was earlier in the VR group (*p* = 0.029). In contrast, the post-intervention activation time of the MF was significantly delayed in the VR group (*p* = 0.001). The IEMGs of TrA (*p* = 0.002) and TA (*p* = 0.007) during CPA1 significantly decreased only in the VR group after the intervention. The VAS scores of three group participants showed significant decreases after intervention (*p* < 0.001).

**Conclusions:**

Patients with CNLBP showed reciprocal muscle activation patterns of the TrA and MF muscles after VR training. VR training may be a potential intervention for enhancing the APAs of the patients with CNLBP.

## 1. Introduction

Chronic nonspecific low back pain (CNLBP) is one of the most common musculoskeletal disorders worldwide [1]. CNLBP may lead to a poor quality of life and increase the economic burden [2]. In recent years, altered postural control patterns have been reported as one of the most important factors that may contribute to CNLBP development [3, 4]. Postural control can be attributed to two different neuromuscular control mechanisms: anticipatory postural adjustments (APAs) and compensatory postural adjustments (CPAs). APAs are feed-forward adjustments that occur before perturbation to minimize the effects of predictable perturbations [5], whereas CPAs are reflexive adjustments made to maintain equilibrium after the onset of perturbations [6]. The central nervous system (CNS) employs APAs and CPAs to maintain stability. Previous studies have reported delayed or absent trunk muscle activation in rapid arm movement or in response to sudden loading in patients with CNLBP [3, 7–9]. For instance, when a resistance force at the back was suddenly released, patients with chronic low back pain (CLBP) showed delayed activation time of abdominal muscles (transverse abdominis (TrA)) in the APA phase in comparison with healthy controls [9]. These findings were supported by the pain adaptation model, which postulates that long-term pain would decrease muscle activation when the muscle is activated as an agonist. Since upper limb loading produces a flexion moment, the TrA is the agonist during sudden upper limb loading and may show decreased electrical activity in the low back pain (LBP) group [9]. The impaired APAs in patients with CNLBP could activate trunk muscles in response to sudden perturbations over time and subsequently lead to the recurrence of LBP. Thus, a training program aimed at enhancing APAs is crucial for CNLBP patients.

Motor control exercise (MCE) is a common exercise rehabilitation program for patients with CNLBP [10]. However, the influence of MCE on APAs in patients with CNLBP is inconsistent. Several studies have reported that MCE could not enhance APA capacity [11–13], while Tsao and Hodges found that MCE can cause early muscle activation [14]. However, the muscle activation time after MCE was later than the time window between -100 and 50 ms, suggesting that MCE may not be a suitable rehabilitation training to enhance the APAs of CNLBP patients and that it may be more beneficial in enhancing postural stability by strengthening muscle power and endurance rather than increasing feed-forward control (e.g., APAs) by the CNS [11, 13].

Many studies have shown that virtual reality- (VR-) based training could improve participants' balance ability by providing multisensory feedback, for example, in chronic poststroke survivors [15, 16] and elderly people [17]. Two previous studies found that VR training could enhance APA capacity [18, 19]. Ida et al. found that when healthy participants lifted one foot to avoid a real or virtual obstacle, muscle activation of the supporting leg and trunk was observed during APAs in both real and VR environments, even though muscle activation in the virtual display setting was smaller than that in the real setting. In another study, PD （Parkinson's disease） participants showed shorter movement time and higher peak velocity of arm movements and longer APAs while catching a fast ball than they did while catching a slow ball in a VR environment. These results suggest that VR-based training can improve APAs. VR training has also been gradually applied to rehabilitation training programs in patients with CNLBP, and it has been shown to reduce pain and improve dysfunction [20]. However, the effect of VR training on APAs in patients with CNLBP is still unknown.

The present study is aimed at investigating the effect of VR-based training on APAs in patients with CNLBP by employing a ball-hitting test. We hypothesized that in comparison with MCE, VR-based training could enhance the APA capacity, including the muscle activation time and integrals of the EMG activities (IEMGs), in patients with CNLBP. The findings of the present study could help verify whether VR training is an effective treatment for enhancing postural control in patients with CNLBP.

## 2. Methods

### 2.1. Participants

Thirty-four right-handed participants with CNLBP were recruited for this study. The inclusion criteria for CNLBP participants were as follows: (1) age between 18 and 40 years, (2) persistent or periodic LBP for longer than 3 months, and (3) no referred symptoms of radiating pain below the knee or paresthesia during the straight-leg raise test. The exclusion criteria were as follows: (1) history of pelvic or spinal column surgery in the past two years; (2) diagnosis of any specific lumbar pathological condition (such as lumbar tumors, vertebral fractures, lumbar spinal stenosis, lumbar spondylolisthesis, rheumatoid arthritis, or ankylosis) and/or severe or progressive scoliosis; (3) body mass index (BMI) ≥ 30 kg/m^2^; (4) history of a treatment program within the past three months; (5) pregnancy; (6) history of severe dysfunction of vital organs (heart, lungs, and kidneys) and/or cognitive deficits; and (7) history of visual or hearing problems. The participants could withdraw the experiment at any time if they (1) were unwilling to participate in this experiment, (2) felt any aggravation of pain during the treatment, (3) had any other disease due to the treatment, and (4) could not complete the proposed treatment plan. Ethical approval for this study (ethics: no. [2020]476) was obtained from the First Affiliated Hospital of Sun Yat-sen University. Written informed consent was obtained from all the participants prior to the experiment.

In our pilot study, the effect sizes (*η*^2^_p_) for the within factor (time) and within-between interaction (time × group) of muscle activation time were achieved in TrA muscle (0.113 and 0.244, respectively). G∗Power (v 3.1.9.7, Germany) was employed to calculate the sample size. 30 participants were required to achieve the statistical power in TrA.

### 2.2. Apparatus and Data Preprocessing

#### 2.2.1. The Ball-Hitting Test

**Figure 1:**
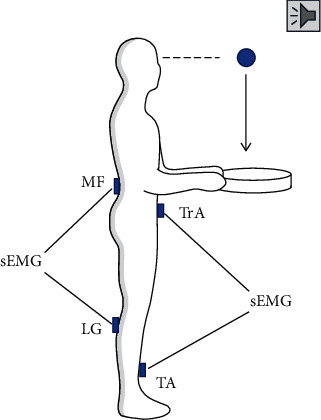
The setting of the ball-hitting test. sEMG: surface muscle electromyography; TrA: transverse abdominis; MF: multifidus; LG: lateral gastrocnemius; TA: tibialis anterior; VR: virtual reality training; MCE: motor control exercise.

The participants stood at the center of a platform with their feet shoulder-width apart. They were asked to keep their elbows bent at 90° while holding a metal tray in their hands. A pressure sensor at the outer center of the base of the tray was used to determine the time point (T0) when the object landed on the tray. After a sound, a load weighing 1.5 kg was suddenly released by the experimenter from the participants' eye level above the tray (Figure 1). The participants were asked to try their best to maintain their body stability throughout the experiment. Each participant repeated five trials with a rest period of approximately 30 s between trials. Surface electromyography (sEMG) data were simultaneously recorded during the ball-hitting test. LabView 15.0.1 software (National Instruments, Austin, TX, USA) was used to simultaneously collect the data from the pressure sensor and the sEMG system at a frequency of 1000 Hz. Sufficient test-retest reliability for five trials in the ball-hitting test was observed in all the muscle activation time and IEMGs (intraclass correlation coefficient: 0.430~0.856).

#### 2.2.2. Surface Electromyography (sEMG)

An sEMG system (Myomonitor IV; Delsys, USA) with eight channels was used to record muscle activity. The interelectrode distance of each channel was 10 mm. Before electrode attachment, the skin of the target areas was prepared by shaving, scrubbing with fine sandpaper, and rubbing with 75% alcohol to reduce impedance. Two electrodes were placed in a vertical arrangement along the muscle fibers of the transverse abdominis (TrA, 2 cm medial and inferior to the anterior superior iliac spine), multifidus (MF, at the level of the L5 spinous process on a line from the posterior superior iliac spine to the interspace between L1 and L2), lateral gastrocnemius (LG, at 1/3^rd^ of the line from fibular head to the lateral side of the Achilles tendon insertion), and tibialis anterior (TA, at 1/3^rd^ on the line between the tip of the fibula and the tip of the medial malleolus). The positions of the electrodes for these four muscles were based on previous studies [7, 8]. The reference electrode was placed on the patella on the dominant side. The sEMG data were sampled at a rate of 1000 Hz.

#### 2.2.3. sEMG Data Preprocessing

The sEMG signals were processed using MATLAB software (The MathWorks Inc., Natick, MA, USA). The raw sEMG signals were rectified and bandpass-filtered (30–400 Hz). The first instance when the pressure signal acquired from the pressure sensor was equal to or greater than 5% of the peak magnitude for at least 20 ms continuously was considered as time zero (T0 = 0, the onset of a rise in the signal of the pressure sensor). The sEMG signals in the ball-hitting test were aligned using T0. The muscle activation time for each trial was detected in a time window from -300 ms to +200 ms in relation to T0. The previously described common methods for detecting muscle activation were not suitable in the present study because the low signal-to-noise ratio (SNR) of the sEMG signals increased the difficulty in detecting the muscle activation time. When the SNR of sEMG signals is very low, the onset time cannot be easily determined by visual inspection of the signal or by simply setting an amplitude threshold [21]. The TKE operation, which simultaneously considers the amplitude and instantaneous frequency of the surface EMG, has been previously used for detection of muscle activation time [21–23]. Thus, in the present study, the TKE operation was applied to determine the onset time of muscle activity. IEMGs were calculated in four epochs, each of 150 ms duration in relation to T0 [5]. The time windows for these four epochs were as follows: (1) from -250 ms to -100 ms (anticipatory reaction, APA1); (2) -100 ms to +50 ms (anticipatory reaction, APA2); (3) +50 ms to +200 ms (early compensatory adjustment, CPA1); and (4) +200 ms to +350 ms (late compensatory adjustment, CPA2). All IEMGs were corrected by the baseline IEMGs from -600 ms to -450 ms relative to T0. The mean muscle activation times and IEMGs were used in the following data analysis.

### 2.3. Interventions

All participants were randomly assigned to three groups: the VR, MCE, and control groups. Participants in the control group received conventional thermal magnetic therapy only, which was performed for 20 min with a medium heat level per day. In addition to thermal magnetic therapy, the participants in the VR group received VR training, whereas those in the MCE group performed MCE. All interventions were performed for two weeks, five days per week. More details regarding VR training and MCE are described below. Three physiotherapists responsible for intervention training were blinded to participants' outcome measures. Each of them took charge of the training program of one group. Two investigators were responsible for demographic information collection and outcome measures, respectively. These two investigators were blinded to the intervention allocation of each participant.

#### 2.3.1. VR Training

**Figure 2:**
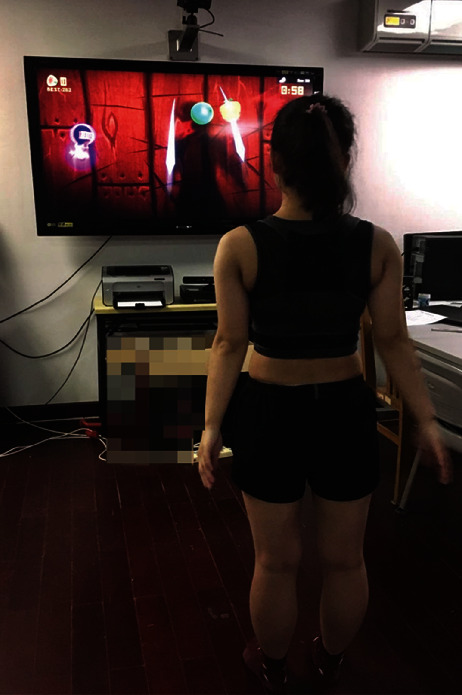
Example of VR training using Kinect Xbox 360 system.

The “Fruit Ninja” game displayed by the Kinect Xbox 360 system was employed for VR training. During the entire training session, the participant stood with feet shoulder-width apart at a distance of 1.5 m in front of the screen. The participants were asked to crush the fruit by waving their hands as much as possible, while simultaneously trying their best to avoid the “bombs” in the game (Figure 2). During training, the participants were asked to avoid trunk bending or turning. The participants needed to complete six sessions of VR training per day. Each session lasted three minutes, with a break of 2 min between sessions. It took approximately half an hour to complete the VR training per day.

#### 2.3.2. Motor Control Exercise (MCE)

The abdominal drawing-in maneuver (ADIM), which is a key technique in MCE training, was designed to enhance coactivation of the TrA and MF to stabilize the trunk before body movement. The ADIM in the present study was based on that used in a previous study [24]. Before the training, the participant learned how to specifically activate the TrA muscle under the guidance of real-time ultrasound without obvious contraction of the internal oblique and external oblique muscles simultaneously. When the participant could perform the ADIM appropriately, MCE training began. The first step of MCE involved three sets of ultrasound-guided ADIM training per day, with a short break of approximately 2 min between sets. Each set involved 10 repetitions of the ultrasound-guided ADIM, each of which lasted for 10 s. In the second step, the participant completed a four-point kneeling exercise. In the second step, the participant completed a four-point kneeling exercise. In the first stage, the participant was instructed to lift one arm with the elbow and wrist extended for 5 s and maintain the TrA contraction at the same time. Each side of the upper limb was repeated thrice with a break of 15 s. In the second stage, the participant lifted one leg with the hip and knee extended for 5 s in a four-point kneeling position. Each side of the leg was repeated thrice with a break of 15 s. In the last stage, the participants raised one arm and the contralateral leg to a horizontal position (bird dog) and held it for 5 s in a four-point kneeling position. Each movement was repeated three times with a break of 15 s. In the last stage, the participants raised one arm and the contralateral leg to a horizontal position (bird dog) and held it for 5 s in a four-point kneeling position. Each movement was repeated three times with a break of 15 s. Each participant was instructed by an experienced physiotherapist during the MCE training. The MCE training required approximately 30 min per day after conventional thermal magnetic therapy.

### 2.4. Procedure

**Figure 3:**
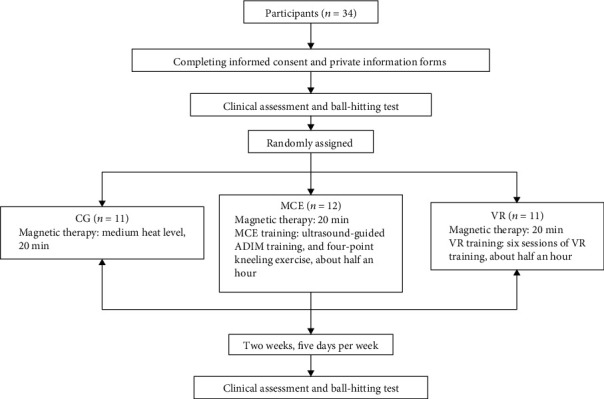
The experiment flowchart. VR: virtual reality training; MCE: motor control exercise; CG: control group; ADIM: abdominal drawing-in maneuver.

Before the training, the participants completed a demographic information questionnaire assessing gender, height, weight, age, and medical history. A visual analogue scale [25] (VAS) (anchored with “painless” at 0 and “intolerable pain” at 10) was used to measure pain intensity, and the Oswestry disability index (ODI) [26] was used to assess function disability pre- and posttraining. The ball-hitting test with sEMG recording was also conducted pre- and postintervention. VR-based training and MCE training were performed after conventional thermal magnetic therapy. The experiment flow is shown in Figure 3.

### 2.5. Statistical Analysis

An independent *t*-test was used to compare between-group differences in all demographic variables except sex (Table 1). The chi-squared test was used to compare between-group differences in sex. The muscle activation times and IEMGs of the four target muscles as well as data from the clinical assessments (including VAS and ODI scores) were analyzed using the two-way mixed-design repeated-measure analysis of variance (ANOVA) with a between-subject factor of group (CG, MCE, and VR groups) and a within-subject factor of time (pre- and posttraining). Post hoc pairwise comparisons were applied when a significant effect was observed. The significance level was set at *p* < 0.05. SPSS software (version 23.0; IBM, Armonk, NY, USA) was used for data analysis.

## 3. Results

### 3.1. Demographics and Clinical Assessments

The demographic information of all participants is shown in Table 1. No between-group differences were found in sex, age, weight, height, BMI, or pain duration (*p* > 0.050) (Table 1).

### 3.2. Muscle Activation Time

**Figure 4:**
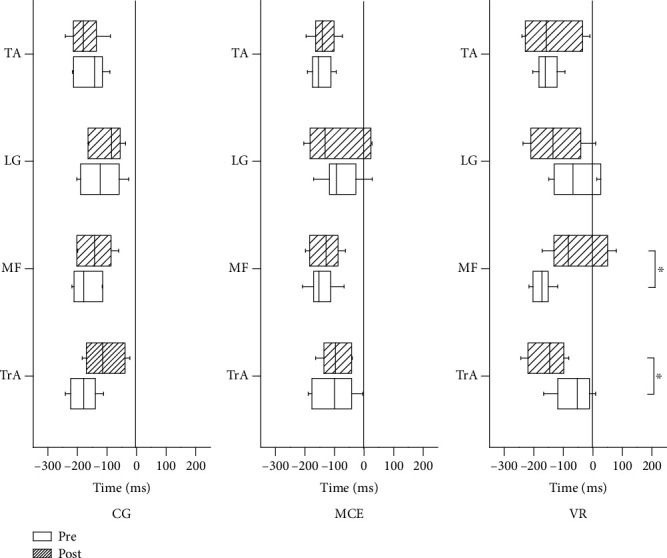
The mean muscle activation times of the four muscles pre- and posttraining for the three groups of participants. TrA: transverse abdominis; MF: multifidus; LG: lateral gastrocnemius; TA: tibialis anterior; VR: virtual reality training; MCE: motor control exercise; CG: control group. ^∗^*p* < 0.050. Standard deviation bars are shown.

The activation times of TrA, MF, LG, and TA are presented in Figure 4. A significant main effect of time was observed on the MF activation time (*F*(1, 31) = 9.438, *p* = 0.004, *η*^2^_p_ = 0.233). A significant interaction effect of time × group was observed on the activation time of TrA (*F*(2, 31) = 4.606, *p* = 0.018, *η*^2^_p_ = 0.029) and MF (*F*(2, 31) = 3.662, *p* = 0.037, *η*^2^_p_ = 0.191). Post hoc analysis for the significant interaction effect revealed that the activation time of TrA after VR training was significantly earlier than that before training (*p* = 0.029). However, the activation time of the MF muscle in the VR group after training was significantly delayed (*p* = 0.001) in comparison with that before training. No changes in the activation times of TrA and MF were observed in the MCE (TrA: *p* = 0.878; MF: *p* = 0.832) and control groups (TrA: *p* = 0.055; MF: *p* = 0.243). Other main effects of time and group and the interaction effect of time × group were not significant (*p* > 0.050).

### 3.3. IEMGs of the Four Muscles

**Figure 5:**
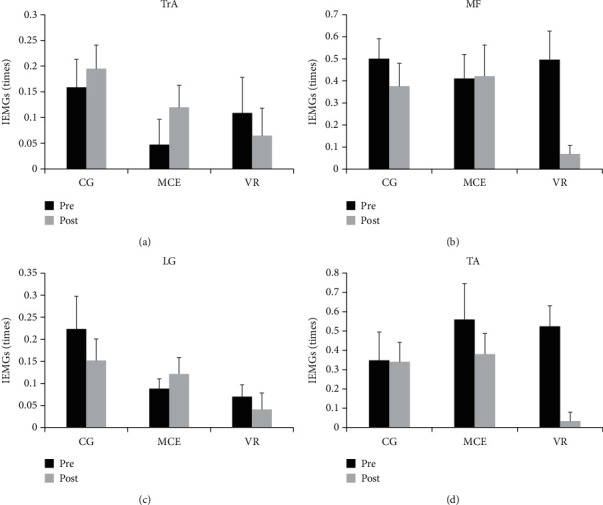
The pre- and posttraining IEMGs of the four muscles during APA1 in the three groups of participants. TrA: transverse abdominis; MF: multifidus; LG: lateral gastrocnemius; TA: tibialis anterior; VR: virtual reality training; MCE: motor control exercise; CG: control group. ^∗^*p* < 0.050. Standard error of the mean bars are shown.

**Figure 6:**
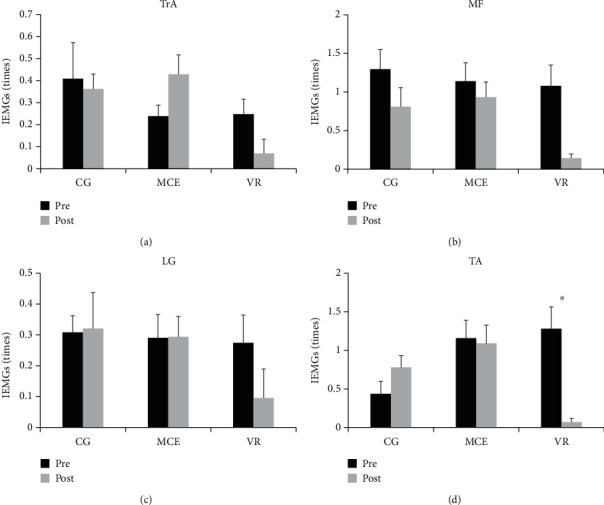
The pre- and posttraining IEMGs of the four muscles during APA2 in the three groups of participants. TrA: transverse abdominis; MF: multifidus; LG: lateral gastrocnemius; TA: tibialis anterior; VR: virtual reality training; MCE: motor control exercise; CG: control group. ^∗^*p* < 0.050. Standard error of the mean bars are shown.

The IEMGs in the APA1 and APA2 phases are presented in Figures 5 and 6, respectively. The *F* ratios and *p* values for the mixed model of the four muscles during APA1 and APA2 are shown in Table 2. A significant effect of time was observed on the IEMGs of MF (*F*(1, 31) = 5.226, *p* = 0.029, *η*^2^_p_ = 0.144) and TA (*F*(1, 31) = 6.404, *p* = 0.017, *η*^2^_p_ = 0.171) during APA1 and on the IEMGs of MF (*F*(1, 31) = 8.344, *p* = 0.007, *η*^2^_p_ = 0.212) and TA (*F*(1, 31) = 0.372, *p* = 0.027, *η*^2^_p_ = 0.148) during APA2. We also observed a significant effect of group on the IEMGs of LG (*F*(1, 31) = 4.243, *p* = 0.024, *η*^2^_p_ = 0.215) during APA1. A significant time × group interaction effect was only observed on the TA during APA2 (*F*(2, 31) = 11.514, *p* < 0.001, *η*^2^_p_ = 0.426). Other main effects of time and group or the interaction effect of time × group were not significant (*p* > 0.050). Post hoc analysis for the significant interaction effect showed that the IEMGs of TA during APA2 were significantly decreased after the intervention only in the VR group (*p* < 0.001), which could not be observed in the other two groups.

**Figure 7:**
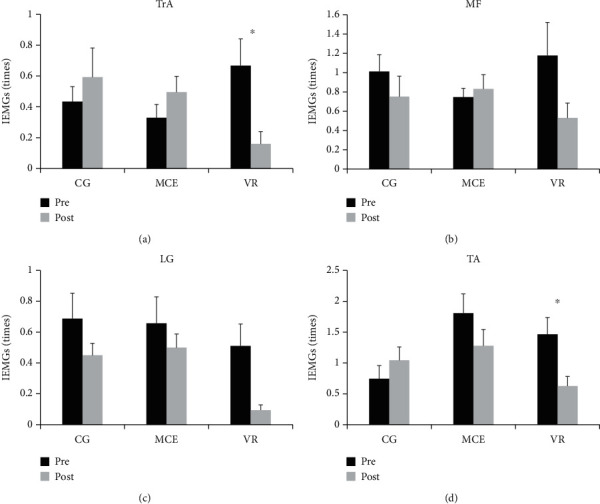
The pre- and posttraining IEMGs of the four muscles during CPA1 in the three groups of participants. TrA: transverse abdominis; MF: multifidus; LG: lateral gastrocnemius; TA: tibialis anterior; VR: virtual reality training; MCE: motor control exercise; CG: control group. ^∗^*p* < 0.050. Standard error of the mean bars are shown.

**Figure 8:**
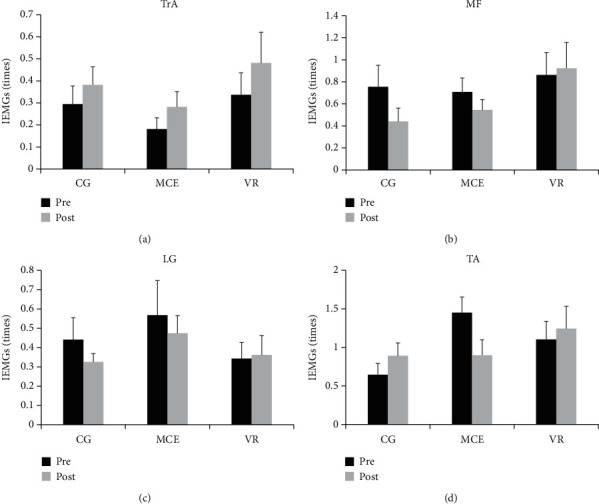
The pre- and posttraining IEMGs of the four muscles during CPA2 in the three groups of participants. TrA: transverse abdominis; MF: multifidus; LG: lateral gastrocnemius; TA: tibialis anterior; VR: virtual reality training; MCE: motor control exercise; CG: control group. ^∗^*p* < 0.050. Standard error of the mean bars are shown.

The IEMGs in the CPA1 and CPA2 phases are presented in Figures 7 and 8, respectively. The *F* ratios and *p* values for the mixed model of the four muscles during CPA1 and CPA2 are shown in Table 3. A significant effect of time was observed on the IEMGs of MF (*F*(1, 31) = 4.256, *p* = 0.048, *η*^2^_p_ = 0.121), LG (*F*(1, 31) = 6.907, *p* = 0.013, *η*^2^_p_ = 0.182), and TA (*F*(1, 31) = 4.589, *p* = 0.040, *η*^2^_p_ = 0.129) during CPA1. A time × group interaction effect was observed on the IEMGs of TrA (*F* (2, 31) = 6.409, *p* = 0.005, *η*^2^_p_ = 0.293) and TA (*F*(2, 31) = 4.103, *p* = 0.026, *η*^2^_p_ = 0.209) during CPA1. Other main effects of time and group and the interaction effect of time × group were not significant (*p* > 0.050). Post hoc analysis for the significant interaction effect showed that the IEMGs of TrA (*p* = 0.002) and TA (*p* = 0.007) during CPA1 significantly decreased after the intervention only in the VR group, which could not be observed in the other two groups.

### 3.4. Pain-Related Clinical Outcomes

Pain-related clinical outcomes, including VAS and ODI scores, are shown in Table 4. A significant main effect of time was observed on the VAS score (*F*(1, 31) = 39.65, *p* < 0.001, *η*^2^_p_ = 0.561). The main effect of group (*F*(2, 31) = 1.26, *p* = 0.298, *η*^2^_p_ = 0.075) and the time × group interaction effect (*F*(1, 31) = 2.013, *p* = 0.151, *η*^2^_p_ = 0.115) were not significant for the VAS score. Time (*F*(1, 31) = 1.70, *p* = 0.203, *η*^2^_p_ = 0.052), group (*F*(2, 31) = 1.19, *p* = 0.317, *η*^2^_p_ = 0.071), and time × group (*F*(1, 31) = 0.023, *p* = 0.978, *η*^2^_p_ = 0.001) did not show significant effects on ODI scores.

## 4. Discussion

The present study investigated the effect of VR training on postural control in patients with CNLBP through measurement of APAs and CPAs in an external postural perturbation task. A novel finding was that the TrA muscle showed earlier muscle activation in APAs after VR training, whereas the MF muscle showed delayed activation after VR training. These findings were not observed in the other two groups. These findings suggest that VR-based training is likely to improve the APAs of CNLBP patients.

Postural control requires the central nervous system (CNS) to receive and integrate multisensory inputs (including vision, vestibular sense, and proprioceptive and tactile information) and thereby coordinate and control the postural muscles to maintain balance and stability of the body [27]. Impaired postural control has been reported to involve APAs and CPAs of trunk muscles [28, 29]. Most previous studies reported that delayed activation of abdominal muscles, especially TrA, in patients with CNLBP was commonly observed in patients with LBP in the postural control assessment, which may increase the recurrence of LBP in patients with CNLBP [4, 30, 31]. The potential reason was that the delayed muscle activation time of TrA in the APAs of patients with CNLBP was associated with remodeling of the motor cortex [32]. Sadeghi et al. found that the prefrontal cortex is activated in patients with LBP during postural interference [33], suggesting that the prefrontal and motor cortices are involved in anticipatory processing.

In this study, the muscle onset time of TrA in CNLBP patients through two weeks of VR exercise training was significantly earlier than that before training, suggesting that VR training could improve APAs. These findings are supported by the results of previous studies [18, 19]. For example, Su et al. showed that training for a ball-catching task in a VR environment could improve PD patients' ability to perform APAs. The possible mechanism is likely that VR training enhanced the activation of the frontoparietal and sensorimotor networks by providing visual cues and visual feedback to patients [34, 35]. The information provided by the virtual environment can increase the activation of the frontal lobe, which participates in the anticipatory process [34]. The anticipation process is a top-down cognitive process [33, 34], the preparatory state of which could be comparable to that during APAs [35]. Thus, in the present study, VR training could improve the APA capacity of CNLBP patients because the visual cues in the dynamic environment of “Fruit Ninja” elicited the participants' anticipation of the movement in response to the objects in the virtual environment. In addition, the “Fruit Ninja” game required the participant to move the arm rapidly in different directions to cut the fruits. Hodges reported that healthy participants would activate TrA muscle earlier than other muscles to maintain postural stability, when performing the rapid shoulder flexion, abduction, extension [36]. The role of TrA muscle in the arm movement in these studies was like to increase the stiffness of the lumbar spine through raising the intra-abdominal pressure and increase the tension of thoracolumbar fascia. Thus, the participants were required to activate TrA earlier to maintain postural stability during the arm movement of the “Fruit Ninja” game.

A previous study reported opposite muscle activation patterns of TrA (delayed activation) and MF (earlier activation) in patients with CNLBP [36]. This reciprocal activation pattern in the TrA-MF pair may be related to an efficient strategy for postural control by the CNS when the perturbation is predictable [37]. The present study showed that the activation of MF was delayed after two weeks of VR training. These results suggest that after VR training, the activation pattern of the MF muscle appears to be similar to that of the control participants.

This study also found that muscle activity of the TA during APA2 was weakened after VR training. A previous study reported that patients with CLBP adopted a body-and-trunk–stiffening strategy and relied more on ankle proprioception to control their posture while standing due to the weakness of their trunk muscles [38]. In this study, the IEMGs of the TA during APA2 decreased after VR training, possibly due to the improvement in TrA muscle activation after VR training. Thus, the improvement in the TrA muscle may enable CLBP patients to achieve better coordination of the deep trunk muscle to maintain stability and rely less on the ankle strategy.

The present study also found that the muscle activities of the TrA and TA in the CPA1 stage were weakened after VR training. These results suggest that patients with CLBP tend to use the APA strategy to reduce the compensatory response of the muscle to external interference after VR training. These findings are consistent with those reported by Santos et al. [5] and Liang et al. [37]. Santos et al. found that when perturbations were predictable, stronger APAs were significantly related to smaller compensatory activities of muscles and COP displacements in response to external perturbation. The findings in Liang et al.'s study showed that after auditory training, the participants demonstrated stronger APAs and less demands on CPAs. In the present study, the IEMGs of TrA and TA of CNLBP participants decreased in CPA1 after VR training, which may result from the APA improvement due to the prediction elicited by the visual information in the virtual environment.

The muscle activation times and IEMGs showed no changes pre- and posttraining in the MCE and control groups. These results were supported by the findings reported by Vasseljen et al. [11] and Lomond et al. [12]. Vasseljen et al. found no significant difference in the activation time of abdominal muscles in patients with CLBP between pretraining and after 8 weeks of MCE training. A planned secondary analysis conducted by Lomond et al. revealed that low back stabilization or movement system impairment treatments did not ameliorate the CLBP participants' APAs impairment, as reflected by the lack of significant effects of treatment on IEMGs. This may be because MCE can enhance postural stability mainly by strengthening muscle power and endurance [12, 13]. However, APAs rely more on brain activities, including motor planning and the excitability of the motor cortex, to predict external perturbations [35, 39]. Thus, in the present study, MCE and conventional physiotherapy did not improve muscle activation time in patients with CNLBP, because the activities in these two groups did not require the participants to perform accurate predictions during the intervention.

As for the clinical outcomes, the VAS findings suggested that LBP decreased after training in the three groups of participants, but there were no statistical differences in VAS scores among the three groups. The improvement in dysfunction revealed by ODI scores was also not significantly different after training among the three groups. Virtual walking was reported to help reduce pain and kinesiophobia and improve function in the short term in patients with chronic nonspecific LBP [40]. The potential mechanism underlying these findings is that the immediate multisensory feedback provided by VR training can improve pain processing in the CNS [41, 42]. The findings in the MCE group are supported by the results of previous studies [36, 43, 44], which showed that MCE could reduce pain and improve dysfunction in CNLBP patients [45], but the clinical improvements did not statistically differ from other treatments [36, 43, 44]. The potential reason for no between-group differences in the pain-related clinical outcomes of the present study was short-term intervention, which may be not long enough to elicit differential treatment effect. The duration of MCE intervention period of 12 weeks was reported to induce superior effect than general exercise program [46]. However, for the duration of the MCE program of 6 weeks, no significant difference was reported between MCE and generally exercise program [47]. Thus, no firm conclusion could be drawn on the comparison of the effectiveness of each intervention program on pain-related clinical outcomes. VR-based intervention may potentially be a beneficial adjunct to MCE intervention for LBP rehabilitation. Further investigation on VR-based training is at least warranted.

### 4.1. Limitations

There are several limitations to the present study. First, sample size was calculated only based on the core muscle of TrA rather than other muscles, which may reduce the statistical power of other muscles. The present study was a preliminary study to explore the effect of VR training on postural control. Large trial is required in the future study. Second, the age range of the sample population was only 19–30 years. Thus, the results may not be directly generalizable to participants beyond this age range. Third, the intervention period was only two weeks, which was potentially not long enough to elicit the treatment effect. Future studies should lengthen the training period to six or more weeks to further confirm the effect of VR training on APAs and pain-related clinical outcomes. In addition to assessments of behavioral outcomes, brain imaging and electrophysiological techniques could be employed to investigate the underlying neural mechanisms in future research.

### 4.2. Conclusions

VR-based training may be an alternative to MCE to enhance APAs by altering the muscle activation pattern of the trunk and lower limb muscles in response to perturbation. The results of this study provide a new potential treatment for APA impairment in CLBP. However, the effect of VR training on the clinical pain symptoms requires further work to verify. In addition to sEMG, future studies can use electrophysiological and brain imaging methods to investigate the underlying neural mechanisms for the effects of VR training on postural control.

## Figures and Tables

**Table 1:** Demographic information of the three groups of participants.

	CG (*n* = 11)	MCE (*n* = 12)	VR (*n* = 11)	*p*
Sex (male/female)	4/7	2/10	3/8	0.563
Age (years)	25.36 ± 3.72	23.75 ± 4.09	21.91 ± 2.43	0.085
Weight (kg)	61.82 ± 9.21	58.58 ± 12.29	58.01 ± 13.29	0.715
Height (m)	1.66 ± 0.07	1.67 ± 0.09	1.67 ± 0.07	0.850
BMI (kg/m^2^)	22.33 ± 2.41	20.70 ± 3.034	20.44 ± 3.54	0.295
Pain duration (months)	49.82 ± 83.49	38.83 ± 37.20	30.18 ± 19.85	0.693

Notes: (1) values are mean ± SD; *n* represents sample size; (2) VR: virtual reality training; MCE: motor control exercise; CG: control group; BMI: body mass index.

**Table 2:** Results of two-way mixed-design ANOVA for IEMGs of the four muscles in APA1 and APA2.

Muscle	APA1						APA2					
	Time		Group		Time × group		Time		Group		Time × group	
	*F* value	*p* value	*F* ratio	*p* value	*F* value	*p* value	*F* value	*p* value	*F* value	*p* value	*F* value	*p* value
TrA	0.382	0.541	1.251	0.300	0.851	0.437	0.021	0.886	2.406	0.107	2.713	0.082
MF	5.226	0.029	0.805	0.456	2.739	0.080	8.344	0.007	2.128	0.136	1.387	0.265
LG	0.259	0.614	4.243	0.024	0.659	0.524	0.338	0.565	1.106	0.343	0.471	0.629
TA	6.404	0.017	0.918	0.410	2.236	0.124	5.372	0.027	2.585	0.092	11.514	<0.001

Note: TrA: transverse abdominis; MF: multifidus; LG: lateral gastrocnemius; TA: tibialis anterior.

**Table 3:** Results of two-way mixed-design ANOVA for IEMGs of four muscles in CPA1 and CPA2.

Muscle	CPA1						CPA2					
	Time		Group		Time × group		Time		Group		Time × group	
	*F* value	*p* value	*F* ratio	*p* value	*F* value	*p* value	*F* value	*p* value	*F* value	*p* value	*F* value	*p* value
TrA	0.599	0.445	0.251	0.780	6.409	0.005	3.474	0.072	1.959	0.158	0.078	0.925
MF	4.256	0.048	0.149	0.862	2.586	0.091	1.113	0.300	2.260	0.121	0.619	0.545
LG	6.907	0.013	2.575	0.092	0.583	0.564	0.601	0.444	1.003	0.379	0.151	0.860
TA	4.589	0.040	2.642	0.087	4.103	0.026	0.130	0.721	1.934	0.162	2.727	0.081

Note: TrA: transverse abdominis; MF: multifidus; LG: lateral gastrocnemius; TA: tibialis anterior.

**Table 4:** The pain-related clinical outcomes in the three groups of participants.

Test	VAS (mean ± SD)			ODI (mean ± SD)		
	CG	MCE	VR	CG	MCE	VR
Pre	3.64 ± 1.36	4.58 ± 1.83	4.36 ± 1.36	12.72 ± 4.84	18.42 ± 9.36	15.65 ± 6.39
Post	2.18 ± 1.17	2.17 ± 1.90	3.18 ± 1.08	9.63 ± 7.20	14.29 ± 21.34	12.77 ± 6.28

Note: CG: control group; MCE: motor control exercise; VR: VR training.

## Data Availability

The EMG datasets analyzed during the current study are available from the corresponding author on reasonable request.
